# Uncooled Microwave Ablation as a Treatment Option to Preserve Thyroid Function in Patients with Benign Thyroid Nodules

**DOI:** 10.5334/jbsr.2746

**Published:** 2022-05-26

**Authors:** Gulsah Yildirim, Hakki Muammer Karakas

**Affiliations:** 1 University of Health Sciences, Istanbul Fatih Sultan Mehmet Training and Research Hospital, Department of Radiology, TR

**Keywords:** Thyroid nodules, thermal ablation, microwaves, thyroid function tests, treatment outcome

## Abstract

**Objective:**

The objective is to evaluate the long-term efficacy and safety of uncooled MWA for the treatment of benign thyroid nodules and its long-term effect on thyroid functions.

**Material and Methods:**

The study was conducted on 40 patients with 40 nodules. They were treated between September 2019 and December 2020. Nodules’ volumes, thyroid functions (triiodothyronine, free thyroxine, thyrotropin) and anti-thyroid peroxidase and anti-thyroglobulin antibodies were measured before treatment and at 3rd, 6th and 12th month following the treatment. Volume reduction rates and changes in clinical findings were evaluated.

**Results:**

The mean volume reduction rate was 49.88, 65.3, and 79.06% at 3rd, 6th, and 12th month, respectively. Antibody levels and thyroid function tests have remained within normal limits and were not exhibited significant change during follow-ups (p > 0.05), except for a significant increase in free thyroxin level at 12th-month (p = 0.007). Subjective symptoms and cosmetic scores were significantly improved all follow-up (p < 0.0001). The only complication was a first-degree skin burn in a patient.

**Conclusion:**

In conclusion, uncooled microwave ablation is an effective and safe method for the treatment of benign thyroid nodules and preserves thyroid function.

## Introduction

Thyroid nodules are very frequent globally, although the majority are benign and asymptomatic. Nevertheless, compressive mass effects and cosmetic problems may develop over time [1,2]. The former comprises the most common treatment indication as it may be symptomatic in a quarter of benign patients [2]. The traditional treatment method for benign thyroid nodules is surgery. However, it has some limitations and a complication rate of up to 10% [3]. Permanent hypothyroidism is also an inevitable end result in total, whereas subclinical or clinical hypothyroidism is the result in subtotal thyroidectomy [4]. Minimally invasive thermal ablation is an alternative to conventional surgery to achieve nodule shrinkage and symptom resolution. Ablation procedures may prevent hypothyroidism in patients as it is targeted to the nodule specifically. Radiofrequency ablation (RFA), the legacy ablation method with accepted efficacy and safety, does not theoretically harm thyroid functions [5,6]. However, there are few cases with transient hyperthyroidism or permanent hypothyroidism after RFA [7,8]. Microwave ablation (MWA) is the newest ablation method for the treatment of benign thyroid lesions with a proven effect on nodule volume and reducing nodule-related symptomatic and cosmetic problems [9,10]. Its long-term effects on thyroid functions and antibodies, however, was not been thoroughly investigated to date. A limited number of studies available had a limited follow-up period or were mostly conducted on cooled systems [11,12].

The objective is to evaluate the long-term efficacy and safety of uncooled MWA for the treatment of benign thyroid nodules and its long-term effect on thyroid functions and antibodies.

## Material and method

### Patients

The study was approved by the Institutional Review Board (Approval no: 17073117_050.06). This study was conducted in accordancewith the Helsinki Declaration principles and Informed consent was obtained for the study.

The retrospective study was conducted on 40 patients who were treated for symptomatic benign thyroid nodule between September 2019 and December 2020. Inclusion criteria were presence of (1) benign diagnosis on two independent Ultrasound (US)-guided fine needle aspiration biopsies (FNAB), (2) solid or mainly solid nodules with solid component >80% on US study, (3) Clinical compressive symptoms or cosmetic concerns and (4) normal thyroid functions. The exclusion criteria were: (1) retrosternal excessive growth, (2) presence of US findings indicating malignant nodule in spite of benign results of biopsies. Nodule volume was not a criterion for inclusion and exclusion.

### Pre-procedural Assessment

All patients were assessed with laboratory tests, US and FNAB. B-mode US was used to evaluate the volume, size and composition of the nodules. Serum levels were determined with electrochemiluminescence immunoassay analyzer (ECLIA, Roche Hitachi Cobas 8000) kits. Laboratory studies included complete blood count, coagulation tests, and complete thyroid function tests (triiodothyronine [T3], free thyroxine [fT4], and thyroid stimulating hormone [TSH] levels). In addition, anti-thyroid peroxidase antibody (anti-TPO) and anti-thyroglobulin (anti-TG) antibody levels were also measured to exclude autoimmune thyroid disease. The reference value ranges accepted as normal in our laboratory are as follows: Anti-TPO: 0–34 IU/ml, Anti-TG: 0–115 IU/ml, TSH: 0.27–4.2 uIU/ml, fT4: 0.93–1.7 ng/dl, T3: 2.04–4.4 pg/ml.

Clinical symptoms were scored by using visual analog scale of 0-10 cm and, a four-point subjective scale was used for cosmetic scoring. According to this scale (I) no palpable mass, (II): an invisible but palpable mass; (III), a visible mass during neck extension or swallowing; and (IV), an easily visible mass.

### Procedure

**Figure 1:**
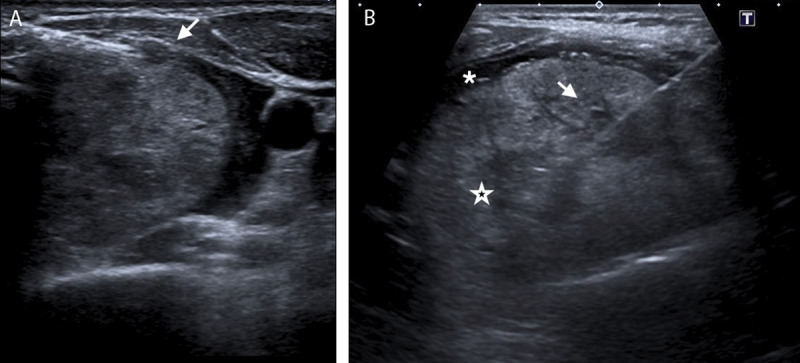
**A.** The local anesthetic mixture was injected to the subcapsular area for hydrodissection and subcapsular anesthesia (arrow). **B.** The transisthmic approach (arrow) was used to visualize the entire length of the antenna and to protect vital structures. Asterisk indicates subcapsular hydrodissection area and star indicates hypoechoic ablation area.

After sterilization of skin and local subcutaneous anesthesia (prilocaine 2%), the hydrodissection technique was performed around the capsule with a mixture (1:1) of 0.5% bupivacaine and 0.9% isotonic NaCl in order to protect the neighboring structures from thermal damage during the procedure (Figure 1A). The MWA system used in this study included a 2.45 GHz microwave generator (TATO, Terumo, Italy) and an uncooled type 18 G, thyroid dedicated probe antenna. The MWA antenna was placed into the nodule along its short axis with the trans-isthmic approach (Figure 1B). Then, 15-watt power was applied with a duration of 10–20 minutes depending on the size of nodules. The procedure was conducted with the ‘multiple overlapping’ technique until the ablation zone was formed in the entire nodule. If the patient couldn’t tolerate the pain associated with procedure, the power was reduced. Possible peri-procedural complications were evaluated by US and clinical symptoms were monitored during the procedure and before discharge. At the end of the procedure, patients remained under observation for two hours and cold compression was applied to the neck to prevent possible hematoma formation.

### Follow-up

**Figure 2:**
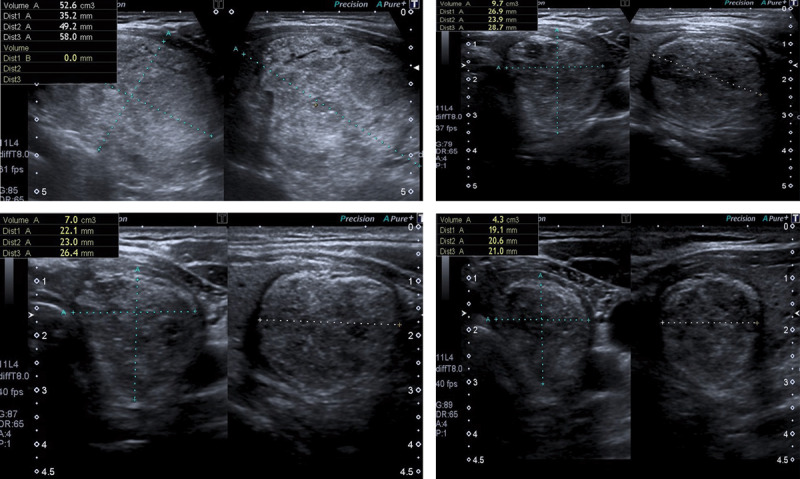
28 years old male patient with a solid nodule in the left thyroid lobe. **A.** Pre-ablative nodule volume was 52.6 ml. **B.** After ablation, the nodule volume decreased to 9.7 ml at the 3rd month follow-up and to 7 ml at the 6th month follow-up **C.** At the end of the 12^th^ month the volume reduced to 4.3 ml **D.**

US examination, laboratory, symptomatic and clinical data were evaluated at 3rd, 6th and 12th month. The US examination was performed to assess the nodule with size, volume and echogenicity (Figure 2). The volume reduction ratio (VRR) was calculated with the following formula: Volume reduction ratio (%) = [(initial volume-final volume) × 100]/initial volume.

### Statistical analysis

Statistical analyzes were performed with IBM SPSS Statistics (version 22, IBM, USA). Data were described using descriptive statistical methods. Continuous variables were reported as the mean ± standard deviation (SD) with range and the categorical variables were reported as frequency and percentage. The compression analyses performed with the Wilcoxon Signed Rank test. All *P* values were reported in an opened form and *P* < 0.05 was chosen as the level of significance.

## Results

There were 24 (60%) males and of 16 (40%) females between 26 and 65 (44.12 ± 10.08) years of age. All the nodules were completely solid. Of them, 18 (45%) were located at right, 16 (40%) were at left and 6 (15%) were at isthmus (Table 1). The primary efficacy outcome was VRR and the other was the preserving thyroid functions. VRRs at 3rd, 6th, and 12th months were 49.88 ± 12.49 (21–82), 65.3 ± 11.78 (34–87), 79.06 ± 8.65 (48–92) %, (p < 0.0001) retrospectively. The mean initial nodule volume was 19.04 ml ± 16.98 and improved significantly at 3rd month (9.55ml ± 11.12, p < 0.0001), 6th month (7.44 ml ± 8.9, p < 0.0001) and 12th month (4.17 ml ± 4.32, p < 0.0001) follow-up. The mean initial largest diameter decreased significantly at follow ups (p < 0.0001) (Table 2).

**Table 1:** Demographic and baseline clinical, hormonal characteristics of patients.


PATIENT’S CHARACTERISTIC’S	

Age, Mean ± SD	47.04 ± 9.34

Gender/Female, n (%)	15/65.2%

Pre-procedural Nodule Volume (ml), Mean ± SD	19.04 ± 16.98

Pre-procedural Longest Diameter (mm), Mean ± SD	39.3 ± 11.3

Nodule Location, n (%)	

Right Lobe	10 (43.5%)

Left Lobe	8 (34.8%)

Istmus	5 (21.7%)

Cosmetic Score, Mean ± SD	3.6 ± 0.49

Symptom Score, Mean ± SD	6 ± 0.73

Hormonal Status, Mean ± SD	

Thyroid Stimulating Hormone (TSH)	1.27 ± 0.64

free Thyroxine (fT4)	1.0 ± 0.13

Triiodothyronine (T3)	3.2 ± 0.38

Antibodies, Mean ± SD	

Anti-thyroidperoxidase (anti-TPO) (IU/ml)	7.5 ± 20

Anti-thyroglobulin (anti-TG) (IU/ml)	5.9 ± 8.4


SD: Standard Deviation.

**Table 2:** Changes of hormonal status, antibodies, volume and clinical findings in follow-up after Microwave ablation.


PARAMETER	PRE-PROCEDURAL	3-MONTH FOLLOW-UP		6-MONTH FOLLOW-UP		12-MONTH FOLLOW-UP	
		
			P VALUE		P VALUE		P VALUE

TSH (uIU/ml)	1.30 ± 0.53	1.46 ± 0.84	0.199	1.45 ± 0.89	0.538	1.46 ± 0.72	0.065

fT4 (ng/dl)	1.07 ± 0.21	1.06 ± 0.20	0.928	1.16 ± 0.23	0.050	1.14 ± 0.21	0.007

T3 (pg/ml)	3.23 ± 0.37	3.27 ± 0.40	0.086	3.26 ± 0.37	0.398	3.17 ± 0.38	0.732

Anti-TG (IU/ml)	6 ± 7	7.29 ± 14.01	0.652	5.19 ± 5.23	0.138	6.96 ± 9.01	0.192

Anti-TPO (IU/ml)	6.05 ± 6.20	6.03 ± 5.92	0.893	6.56 ± 5.94	0.139	7.12 ± 5.72	0.410

Volume (ml)	21.08 ± 15.29	10.63 ± 9.58	<0.0001	7.20 ± 7.19	<0.0001	4.16 ± 3.45	<0.0001

Longest Diameter (mm)	39.35 ± 11.32	33.00 ± 9.02	<0.0001	29.85± 9.14	<0.0001	25.8 ± 8.54	<0.0001

VRR (%)	–	49.88 ± 12.49	<0.0001	65.3 ± 11.78	<0.0001	79.06 ± 8.65	<0.0001

Cosmetic Score	3.52 ± 0.50	2.45 ± 0.84	<0.0001	2.15 ± 0.83	<0.0001	1.92 ± 0.88	<0.0001

Symptom Score	6.25 ± 0.74	2.42 ± 1.19	<0.0001	1.47 ± 0.96	<0.0001	0.95 ± 0.74	<0.0001


Data presented as a mean ± SD (p > 0,05 means there is no difference between the pre-procedure and follow-up.) VRR: Volume Reduction Ratio.

Initial mean of T3, fT4 and TSH were 3.23 ± 0.37 pg/ml, 1.07 ± 0.21 ng/dl, 1.30 ± 0.53 uIU/ml retrospectively. Serum levels of thyroid hormones were in normal limits at the baseline and 3 [(T3 (p = 0.086), fT4 (p = 0.928), TSH (p = 0.199)], 6 [(T3 (p = 0.398), fT4 (p = 0.050), TSH (p = 0.538)], 12 [(T3 (p = 0.732), fT4 (p = 0.007), TSH (p = 0.065)] month follow-up. Although fT4 level remained within normal values, a minimal increase was observed at the 12th-month follow-up compared to the baseline. Free T4 level was increased to 1.14 ± 0.21 from 1.07 ± 0.21 (p = 0.010) Anti-TPO (6.05 ± 6.20) and anti-TG (6 ± 7) levels did not change significantly during 3 [(Anti-TPO (p = 0.893), anti-TG (p = 0.652)], 6 [(Anti-TPO (p = 0.139), anti-TG (p = 0.138)] or 12 [(Anti-TPO (p = 0.410), anti-TG (p = 0.192)] month follow-up. Laboratory parameters remained within normal limits in all patients (Table 2).

The pre-procedural mean symptom score was 6.25 ± 0.74, and it showed a rapid improvement at the 3rd month follow-up and decreased to 1.47 ± 0.96 (p < 0.0001). On the other hand, the mean cosmetic score was 3.52 ± 0.50 and decreased to significantly (p < 0.0001) at the follow-ups (Table 2).

There was no major complication. Minor complication was seen in only 2.5% (n = 1) of patients. That was a first-degree skin burn and resolved spontaneously.

## Discussion

Standard therapeutic procedures for benign thyroid nodules include radioactive iodine and surgery. However, these has some serious complications and also permanent effects on thyroid functions. Thermal ablation techniques have become very popular as they are a less invasive, cost- and risk-effective method than surgery to reduce nodule volume and improve symptoms. Both RFA and MWA improve symptoms and cosmetic findings associated with thyroid nodules as effectively as surgery with lower complication rates [9,13]. Several studies revealed the preservation of thyroid function in RF ablation, but data on the impact of MWA on thyroid function and antibodies are limited and the follow-up period of relevant studies is short (i.e., less than 6 months) [7,11,12,14]. Apart from those clinical results for MWA are comparable with RFA [6,15]. The VRR defined in the literature varies between 40–80%, 48–86, and 75–90% at 3, 6, and 12-month follow-up, respectively [16,17]. In our study nodules were decreased in volume at follow up. In this context, mean VRRs were 50%, 65% and 79% at 3-month, 6-month and 12-month follow-ups, respectively. Although the VRR is within the limits of the literature data, they were at the lower end of it. The reason for that could be the performance of a single ablation session per nodule. Ablation of the peripheral portions of the nodules may have been insufficient in an attempt to avoid complication. Multiple ablations may be beneficial for residual tissue ablation to improve VRR [7].

Several studies showed that RFA is an effective and reliable method in protecting the thyroid gland, with long follow-up periods [7,18]. There are few studies in the literature about the effects of MWA on thyroid function in patients with benign thyroid nodules [11,12,19]. Heck et al. [12] investigated the effects of MWA on thyroid function and antibodies, and they showed that laboratory parameters did not change significantly at the 3-month or 6-month follow-up (p > 0.05). Also, in their study thyroglobulin levels increased within 24 hours after the procedure and decreased at 3-month follow-up (p < 0.05). MWA induces coagulative necrosis in the ablation zone, and it can cause thyroglobulin (Tg) release in thyroid cells. Heck et al. [12] and Erturk et al. [11] investigated its use by associating Tg measurements with volume reduction and did not find any correlation. Also, some investigators suggested that MWA exposure triggers autoimmunity by causing Tg release and leads to an increase in anti-TG levels during the 6-month follow-up [20]. In our study, Tg levels were not measured but thyroid autoimmunity did not develop during 12-month follow-up in anti-Tg and anti-TPO negative patients. In a long-term follow-up (12 month) study with a large sample size, Honglei et al. [19] also evaluated thyroid functions and no significant difference was observed. However, antibody monitoring was not performed in this study.

In this study, uncooled MWA was found to have low complication rates. There was no major complication. Only one patient developed first-degree skin burn that was resolved spontaneously. According to the literature, the most common and serious complication is the recurrent laryngeal nerve injury with an incidence of 0–9.1% rate [17]. We have not encountered this complication possibly due to differences in techniques such as the creation of sleeve with hydrodissection, use of multiple overlapping technique, trans-isthmic approach and meticulous monitoring of the dangerous triangle in which the recurrent laryngeal nerve is located. On the other hand, as a limitation, we didn’t measure the proximity of the ablated nodules relative to the tracheoesophageal groove. The width of the thyroid parenchyma between the nodules we treat and the tracheoesophageal groove may be a risk factor for the development of recurrent nerve damage. Studies on this subject will be beneficial.

## Conclusion

Uncooled MWA helps to improve symptoms and cosmetic scores by decreasing nodules’ volumes, and to preserve normal thyroid function in patients with benign thyroid nodules. This study has revealed long term presence of these findings and supports findings of larger patient series with shorter follow-ups.
